# Myocardial viability in preserved or mildly impaired left ventricular function prior to revascularization - findings from a 3 year experience

**DOI:** 10.1186/1532-429X-17-S1-P133

**Published:** 2015-02-03

**Authors:** Hannah Douglas, Ben Cole, Chin Munn Soong, Paul Horan, Lana Dixon, Nicola Johnston, Mark Harbinson

**Affiliations:** Cardiology, Belfast Heart Centre, Belfast, UK; Queen’s University, Belfast, UK

## Background

Viability testing prior to revascularization in ischaemic cardiomyopathy has courted controversy in the literature over recent years. Viability assessment prior to revascularization at our institution is largely requested for patients with severely impaired left ventricles (LV). This study however aims to review the outcomes of the patients with preserved or mildly impaired LV function who underwent cardiac magnetic resonance imaging (CMR) for viability assessment during consideration for revascularization and to evaluate its use in this group.

## Methods

Patients undergoing CMR scans to assess viability prior to coronary artery revascularization were identified from January 2011 until June 2013. Demographics, viability and outcome data were collected for all.

## Results

Viability assessments were undertaken in 256 patients who were referred for revascularization of coronary artery disease by either percutaneous or surgical methods. Of these patients 71 (27.7%) were found to have preserved or mildly impaired LV function, defined by an ejection fraction greater than 45% (72% male, mean age 66±12.8 years). Adenosine stress perfusion was performed in 25.4%. Ultimately 76% were revascularized (38% underwent coronary artery bypass grafting (CABG) while 38% had percutaneous coronary intervention (PCI)). The remaining 24% were turned down for revascularization. The reason not to revascularize in 11.7% of cases was LAD territory nonviability. Other reasons were independent of the viability findings and included poor target vessels, co-morbidities and patient decision. Mean LV measurements within each group were as follows: ejection fraction CABG 56.67±7.58%, PCI 57.41±8.58%, medical management 59.4±19.65% (p=0.58) LVEDV CABG 87.74±19.37mls, PCI 85.22±20.62mls, medical management 80.41±16.14mls (p=0.47). The number of non-viable segments within each group was: CABG 2.15±1.99, PCI 2.15±2.13 and medically managed 2.41±2.06 (p= 0.90). The nonviable segments were in LAD territory in: CABG 22.2%, PCI 33.3% and medical management 29.4% (p=0.66). Median follow up was 2.4 years. Outcome data was as follows; MACE: CABG 14.8%, PCI 7.4% and medical management 11.8% (p=0.69); death: CABG 11.1%, PCI 7.4%, with no reported deaths in the medical management group (p=0.37); revascularization: CABG 3.7%, PCI 11.1% and medical management 11.8% (p=0.53) and cardiac rehospitalisation: CABG 14.8%, PCI 3.7% and medical management 11.8% (p=0.37).

## Conclusions

LV function and volume measurements and the number and territory of nonviable segments in preserved or mildly impaired left ventricular function was not significantly different between revascularized and medically managed patients. The rates of events or death did not differ significantly. Therefore CMR for viability assessment has not been shown to be a useful tool in guiding revascularization for patients with preserved or mildly impaired ventricles. We propose that stress perfusion without viability would improve the utility of CMR in this group of patients.

## Funding

N/A.

